# Nonclassically Secreted Regulators of Angiogenesis

**DOI:** 10.4172/2329-9495.1000101

**Published:** 2013-02-28

**Authors:** Igor Prudovsky

**Affiliations:** Maine Medical Center Research Institute, 81 Research Drive, Scarborough, ME 04074, USA

**Keywords:** Angiogenesis, Regulator, Nonclassical secretion

## Abstract

Many secreted polypeptide regulators of angiogenesis are devoid of signal peptides. These proteins are released through nonclassical pathways independent of endoplasmic reticulum and Golgi. In most cases, the nonclassical protein export is induced by stress. It usually serves to stimulate repair or inflammation in damaged tissues. We review the secreted signal peptide-less regulators of angiogenesis and discuss the mechanisms and biological significance of their unconventional export.

## Nonclassical Secretion: How and Why Some Secreted Proteins Avoid ER-Golgi on their Exit Way

Angiogenesis, the growth of new vessels from the pre-existing elements of the vascular system is critically important for cardiovascular development, repair of damaged tissues, inflammation and tumor formation. A plethora of secreted proteins participates in the regulation of angiogenesis. Among these polypeptides are growth factors and cytokines, which signal through specific cell membrane receptors. Many extracellular enzymes also modulate angiogenesis.

Various stress conditions influence angiogenesis, particularly by increasing the availability of secreted pro-angiogenic proteins. For example, hypoxia stabilizes the transcription factor HIF1α, which stimulates the expression of Vascular Endothelial Growth Factor (VEGF), the major inducer of angiogenesis [1]. Alternatively, mechanical stretch can also enhance the expression of VEGF [2]. The availability of extracellular proteins can be regulated not only at the level of their expression but also through the modulation of their secretion. Most secreted proteins have in their primary structure a N-terminally located cleavable hydrophobic signal peptide, which is required for the translocation of newly translated polypeptides into the endoplasmic reticulum (ER) followed by the transport through the ER-Golgi compartment and release mediated by the fusion of Golgi-derived exocytotic vesicles with the cells membrane [3]. This export pathway is known as classical protein secretion. Some cells, such as neuroendocrine cells [4] sort classically secreted proteins into constitutive and regulated secretory pathways. In the latter pathway, the fusion of secretory vesicles with the plasma membrane (on which they are poised) is induced by a drastic increase of cytosolic calcium concentration. However, so far there is no evidence of regulated classical secretion of angiogenic regulators. At the same time, many polypeptide regulators of angiogenesis are secreted through ER-Golgi-independent pathways, and secretion of these proteins is often induced by stress conditions. Unlike classically secreted proteins, unconventionally or nonclassically secreted polypeptides are devoid of signal peptides. They are not detected in the ER-Golgi compartment, and their export is not inhibited by brefeldin A, a compound, which blocks the protein transport from the ER to Golgi [5,6].

Nonclassically secreted proteins present important potential targets for the modulation of angiogenesis in the context of inflammation, cardiovascular diseases and cancer. They display non-overlapping functions and distinct, not fully understood mechanisms of secretion. Interestingly, several nonclassically released proteins can perform not only extracellular, but also intracellular, and in particular-nuclear functions. The trafficking through the ER-Golgi would preclude this functional dualism. In addition, the absence of signal peptides in the primary structure of the proteins excludes the unregulated constitutive classical export and thus prevents the potential undesirable effects of secreted proteins. Indeed, many nonclassically released proteins are strong inducers of inflammation, atherogenesis and tumor growth, and their export needs to be strictly controlled. For example, unlike invertebrate FGFs, FGF1 and FGF2, two most ubiquitously expressed members of FGF family, lack signal peptides, which were lost during the evolution [7]. It is noteworthy that the artificial addition of a signal peptide to FGF1 turns it into a potent oncogene [8]. The stress-induced export of FGF1 [9] apparently limits its delivery to specific situations of tissue damage, when it is needed to stimulate the repair processes.

Based on the present knowledge, the nonclassically secreted regulators can be classified into two groups according to the general mechanisms of their release. The first group involves proteins, which are incorporated in an ER-Golgi-independent manner into various cytoplasmic vesicular structures including endolysosomes, autophagosomes and multivesicular bodies. These proteins are exported as a result of fusion of such cytoplasmic organelles with the cell membrane. Another group is represented by proteins which directly translocate through the plasma membrane from the cytosol to the extracellular compartment. However, the mechanisms of the nonclassical secretion of many signal peptide-less proteins still remain unknown.

## Signal Peptide-Less Proteins Released through Vesicular Cytoplasmic Structures

The interleukin 1 (IL1) group of cytokines includes several signal peptide-less nonclassically secreted regulators of angiogenesis [10]. The best-studied member of the family, pro-inflammatory protein IL1β, signals through the Type 1 IL1 receptor (IL1R1) [10]. It promotes angiogenesis in various pathological contexts [11,12]. It is also involved in the transdifferentiation of Vascular Smooth Muscle Cells (VSMC), which occurs during atherosclerosis and restenosis [13,14]. Like other IL1 family members, IL1β is produced as a precursor that undergoes proteolytic maturation. Caspase-1, a component of cytoplasmic multiprotein complexes termed as inflammasomes, cleaves preIL1β and produces mature IL1β that is nonclassically secreted [10]. The maturation and export of IL1β are induced during the activation of Toll-like Receptors (TLR) [15] and this induction requires the intracellular production of Reactive Oxygen Species (ROS) [16]. The release of IL1β has been studied on monocytes, macrophages and dendritic cells. A variety of secretion mechanisms were proposed for IL1β. Rubartelli et al. [16] found that IL1β translocates from the cytosol into endolysosomes (organelles formed as a result of fusion of endosomes with lysosomes), and then it is released upon endolysosome fusion with the cell membrane [17]. Other authors reported a shedding of IL1β-containing microvesicles from the cell membrane [18], export of IL1β in exosomes (vesicles contained in the multivesicular bodies and released during the fusion of the latter with the cell membrane) [19]. Recent publications demonstrate the importance of autophagy [15,20] and Golgi Reassembly Stacking Protein (GRASP) [20] for IL1β secretion. GRASP is an interesting case of a classical secretion pathway element being diverted in the nonclassical protein release [6]. Another such case is the earlier reported participation of a short form of synaptotagmin 1 in the nonclassical export of FGF1 [21] (Table 1).

IL33, which also belongs to IL1 family, undergoes caspase-1-dependent proteolytic maturation [22]. It signals though Interleukin 1 receptor-like 1 (IL1RL1 or ST2) [23]. IL33 is a potent inducer of angiogenesis and vascular permeability [24]. It is localized in both cell nuclei and cytoplasmic vesicles, and mechanical stress induces its release [25].

High Mobility Group Protein 1 (HMGB1) is best known as a nuclear protein, which interacts with transcription factors and histones and participates in the regulation of transcription [26,27]. However, it can also be released through a nonclassical pathway and signal through pattern recognition receptors TLR2 and TLR4 and Receptor of Advanced Glycation End products (RAGE) [28]. Extracellular HMGB1 stimulates tumor angiogenesis [29], promotes neurovascular remodeling after stroke [30] and contributes to pulmonary hypertension [31]. HMBG1 release is induced by stresses such as ischemia [32] and lipopolysaccharide treatment [33]. The nonclassical export of HMGB1 is dependent on the phosphorylation of specific serine residues, which results in its relocation from the nucleus to cytoplasm [34,35]. HMGB1 secretion requires the activation of inflammasomes [36]. Activation of monocytes results in the redistribution of HMGB1 from the nuclei to endolysosome-like vesicular structures, which apparently serve as vehicles for its export [37].

Not only signaling polypeptides but also enzymes can be released through nonclassical pathways. Tissue transglutaminase (tTG) is an extracellular signal peptide-less protein [38]. This enzyme, which catalyzes the cross-linking of extracellular matrix proteins, is involved in many aspects of normal and pathological cardiovascular physiology [39]. It enhances angiogenesis in the process of tissue repair [40], and ani-tTG autoantibodies disturb angiogenesis [41]. The secretion of tTG is apparently mediated by the penetration of tTG inside the forming endosomes, which eventually results in the release of tTG after the fusion of recycling endosomes with the cell membrane [38]. The process of tTG export is negatively regulated by nitric oxide [42].

## Signal Peptide-Less Proteins that Directly Translocate through the Plasma Membrane

Most secreted proteins, which belong to the large Fibroblast Growth Factor (FGF) family, have signal peptides in their primary structure and thus are released through the conventional ER-Golgi-dependent pathway. However, two ubiquitously expressed representatives of the family, FGF1 and FGF2, are devoid of signal peptides and utilize nonclassical pathways for their release. Both of them are diffusely distributed in cytoplasm and not observed in vesicular structures. Their export occurs as a result of direct translocation through the cell membrane [5,6]. FGF1 and FGF2 are major pro-angiogenic proteins, involved in multiple cardiovascular pathologies, inflammation and tumor growth [43,44].

FGF1 secretion is induced by various types of cell stress, such as hyperthermia, hypoxia and growth factor starvation [9,45,46]. It is dependent on the formation of a multiprotein complex, which contains a covalent FGF1 dimer [47] and several other signal peptide-less proteins: S100A13 [48], a 40 kDa form of synaptotagmin 1 [49,50] and the enzyme sphingosine kinase 1 (SphK1) [51]. All members of the complex bind copper ions, and copper is critical for FGF1 export [51,52]. FGF1 destabilizes liposomes composed of acidic phospholipids (pL) [53]. Mutations abolishing acidic PL binding result in a drastic inhibition of FGF1 export [53]. FGF1 is exported through membrane domains, which are characterized by externalization of the acidic PL phosphatidylserine (PS) [54]. Chemical components, which inhibit PS externalization, suppress FGF1 release [54]. Stress-induced PS externalization may serve as a driver of the nonclassical export of FGF1 [54]. Besides cell stress, thrombin treatment [55] and Notch signaling inhibition [56,57] both induce the export of FGF1. Damaged and ischemic tissues are characterized by a wide spectrum of conditions propitious for FGF1 secretion: hyperthermic and hypoxic stresses, proteolytic activation of thrombin and decrease of Notch signaling because of the loss of cell-cell contacts.

Unlike stress-dependent FGF1 secretion, FGF2 export does not necessarily require stress [58]. It depends on PL PIP2, which is localized in the inner leaflet of the cell membrane [59]. FGF2 secretion is dependent on Tyr 82 phosphorylation by Tec kinase [60]. Similar to FGF1, FGF2 destabilizes liposomes by producing pores in artificial PL bilayers [61]. The externalization of FGF2 apparently involves glycosylated cell membrane counter-receptors [62]. The release of both FGF2 [63] and FGF1 [64] does not require their unfolding.

Similar to IL1β, the cytokine IL1α signals through IL1R1. Precursor IL1α exhibits nuclear localization dependent on a specific sequence, which is cleaved during the proteolytic maturation catalyzed by calpain [65]. Secreted IL1α promotes tumor angiogenesis [66]. The knockout of IL1α significantly decreases the formation of atherosclerotic plaques [67]. In addition, IL1α promotes the proinflammatory phenotype of VSMC [68]. The localization of mature IL1α in cytoplasm is diffuse, without signs of association with vesicular structures [69]. Similar to FGF1, its release induced by hyperthermic stress is dependent on copper ions and the signal peptide-less protein S100A13 [69]. Keller et al. [70] reported that although caspase-1 is not involved in IL1α maturation, the lipopolysaccharide-stimulated release of IL1α is drastically reduced in caspase-1 −/− monocytes and keratinocytes.

Galectins, a family of beta-galactose-binding proteins, are devoid of signal peptide and can have both intracellular and extracellular functions [71]. Secreted galectin 1 [72] and galectin 3 [73] enhance tumor angiogenesis. Galectin 1 is a binding target of the angiostatic agent 6DBF7 [74]. Genetic inactivation of galectin 3 gene in the mouse ApoE null model of atherosclerosis resulted in the decrease of atherosclerotic plaque formation [75]. The nonclassical release of galectin 1 depends on its beta galactose-binding domain and apparently on glycosylated cell surface counter-receptors [76]. Similarly to FGF2, galectin 1 can directly translocate into the isolated membrane vesicles [77].

## Signal Peptide-Less Proteins with Yet Undetermined Mechanism of Nonclassical Export

Macrophage Migration Inhibitory Factor (MIF), a signal peptide-less pleiotropic cytokine, signals through the chemokine receptors CXCR2 and CXCR4 [78]. It is involved in atherosclerosis by promoting the recruitment of atherogenic leukocytes [79]. In addition, MIF was recently shown to stimulate the differentiation of endothelial precursor cells into VSMC and endothelial cells [80]. The secretion of MIF is induced by hypoxia [81], and it is dependent on a cell membrane ABC transporter [82]. Interestingly, Golgi-associated p115 protein is involved in MIF export [83].

Small calcium binding proteins of the S100 family perform a variety of intracellular functions [84]. Alternatively, these signal peptide-less proteins can undergo nonclassical secretion and signal through the RAGE receptor [85]. S100A7 (psoriasin) enhances the proliferation of endothelial cells by stimulating VEGF expression through a RAGE-dependent mechanism [86]. S100A4 (metastasin) stimulates angiogenesis [87] and invasive growth of endothelial cells [88].

Annexin 2, a signal peptide-less protein, which forms a heterotetramer with two molecules of S100A10 can be found both inside the cell and on the cell surface [89]. Thrombin treatment induces the externalization of Annexin 2 [90]. When the Annexin 2 tetramer locates at the cell surface, it binds plasminogen and tissue plasminogen activator, thus presenting a platform for plasmin generation [91]. Annexin 2 externalization depends on its Tyr23 phosphorylation [92]. Cell surface Annexin 2 presents a target for peptide inhibitors of angiogenesis TM601 [93] and angiostatin [94]. So far, there is no generally accepted view on the mechanism of Annexin 2 export. While Rabouille et al. [95] classify Annexin 2 as a protein transported through the cell membrane, Valapala and Vishvanatha [96] have demonstrated that it can be externalized in exosomes released after the fusion of multivesicular bodies with the cell membrane.

Endothelial Monocyte-Activating Polypeptide II (EMAP II) is a signal peptide-less pro-inflammatory and anti-angiogenic cytokine [97]. Interestingly, EMAP II precursor has a distinct intracellular function as an auxiliary component of the tRNA multisynthetase complex [97]. Apparently, caspases 3 and 7, MMP-9, elastase, and cathepsin L are involved in the processing of EMAP II from the 43 kDa precursor (p43) to the 23 kDa mature EMAPII form [98,99]. Both precursor and mature EMAP II are equally biologically active and signal through the CXCR3 receptor [100,101]. Both EMAP II forms are nonclassically secreted under conditions of stress including stimulation with cytokines, cigarette smoke and hypoxia [99,102–104]. EMAP II promotes cigarette smoke-induced emphysema and endothelial apoptosis [103,104], induces the migration of endothelial progenitor cells [100], causes the nitric oxide-dependent pulmonary artery dilatation [105], stimulates the opening of the blood-tumor barrier [106] and interferes with VEGF-induced pro-angiogenic signaling [107].

Signal peptide-less enzyme SphK1, which catalyzes the production of a potent pro-angiogenic lipid sphingosine-1-phosphate, exhibits nonclassical secretion [108].

IL18, a member of IL1 family, signals through the specific IL18 receptor [109]. Mature IL18 is released after the cleavage of its precursor by caspase-1 [110]. Depending on the context, the effect of IL18 on angiogenesis can be both positive and negative. Thus, IL18 promotes angiogenesis in the arthritic pannus [109,111] apparently as a result of stimulation of VEGF expression in arthritic fibroblasts [112]. Conversely, IL18 inhibits tumor angiogenesis [113], possibly because it enhances the expression of angiogenesis inhibitor thrombospondin-1 [114].

## Conclusion

Nonclassically secreted proteins form a large and important group of the regulators of angiogenesis. The absence of signal peptides enables many of these proteins to execute both intracellular and extracellular functions. Unlike the constitutive ER-Golgi dependent secretion, nonclassical protein release is highly regulated, particularly by different types of stress. The mechanisms of nonclassical secretion are still far from being sufficiently understood. However, there are at least two major types of nonclassical release: direct translocation through cell membrane (e.g., FGF1 and FGF2) and export mediated by intracellular vesicles of various types (e.g. IL1β). Further understanding of the molecular mechanisms of unconventional secretion is important to regulate the available levels of biomedically important signal peptide-less proteins.

## Figures and Tables

**Table 1:** Nonclassically released vascular regulators.

Nonclassically secreted protein	Vascular effects	Type of nonclassical export
IL1β	Promotes pathological angiogenesis	Vesicular
IL33	Enhances angiogenesis	Vesicular
HMGB1	Enhances tumor angiogenes, promotes neovascular remodeling	Vesicular
Tissue transglutaminase	Enhances angiogenesis	Vesicular
FGF1	Stimulates angiogenesis	Transmembrane
FGF2	Stimulates angiogenesis	Transmembrane
IL1α	Promotes tumor angiogenesis; enhances formation of atherosclerotic plaques	Transmembrane
Galectin 1	Promotes tumor angiogenesis	Transmembrane
Annexin 2	Serves as a platform for plasmin generation on endothelial cell surface	Undetermined
S100A4	Enhances angiogenesis	Unknown
S100A7	Enhances angiogenesis	Unknown
IL18	Can enhance or repress angiogenesis, dependent on the tissue context	Unknown
EMAPII	Inhibits angiogenesis, induces endothelial cell apoptosis	Unknown
SphK1	Stimulates angiogenesis through sphingosine-1-phosphate production	Unknown
